# Diagnosis and management of ADHD: a pediatric perspective on practice and challenges in Switzerland

**DOI:** 10.1186/s12887-023-03873-x

**Published:** 2023-03-04

**Authors:** A. Zysset, D. Robin, K. Albermann, J. Dratva, S. Hotz, F. Wieber, M. von Rhein

**Affiliations:** 1 ZHAW Zurich University of Applied Sciences, Winterthur, Switzerland; 2 Centre of Social Pediatrics, Cantonal Hospital Winterthur, Winterthur, Switzerland; 3 University of Basel, Basel, Switzerland; 4 University Neuchatel, Neuchatel, Switzerland; 5 University of Konstanz, Constance, Germany; 6 University Children’s Hospital Zurich, Zurich, Switzerland; 7 University of Zurich, Zurich, Switzerland

**Keywords:** ADHD, Pediatricians, Guidelines, ADHD diagnosis, ADHD treatment, ADHD management

## Abstract

**Background:**

Attention deficit/hyperactivity disorder (ADHD) is one of the most prevalent psychiatric disorders in childhood. In Switzerland, the complex diagnosis and treatment are being carried out by adolescent−/child psychiatrists, and pediatricians. Guidelines recommend a multimodal therapy for patients with ADHD. However, it has been questioned whether health professionals follow this approach or favor drug therapy. This study aims to provide insights into the practice of pediatricians in Switzerland regarding diagnosis and treatment of ADHD and their perceptions of these processes.

**Method:**

An online survey (self-report) about current practices of diagnosis and management as well as challenges regarding ADHD was distributed to office-based pediatricians in Switzerland. One hundred fifty-one pediatricians participated. Results show that therapy options were almost always discussed with parents and older children. Exchange with parents (81%) and level of child’s suffering (97%) were central when selecting therapy options.

**Results:**

Therapies about which pediatricians informed most often were: pharmacological therapy, psychotherapy, and multimodal therapy. Challenges voiced were the subjectivity of diagnostic criteria and dependence on third parties, low availability of psychotherapy, and a rather negative public attitude towards ADHD. Needs that were expressed were further education for all professionals, support for coordination with specialists and schools as well as improvement of information on ADHD.

**Conclusions:**

Pediatricians do consider a multimodal approach when treating ADHD and take the families` and children’s opinions into account. Improvements of the availability of child and youth psychotherapy, the strengthening of the interprofessional cooperation with therapists and schools, and efforts to increase public knowledge about ADHD are proposed.

## Background

Attention deficit/hyperactivity disorder (ADHD) is one of the most common neurodevelopmental disorders in childhood and youth with a worldwide prevalence between 5 and 8% [1, 2]. ADHD leads to numerous negative outcomes for the affected children with regards to their personal life, their family environment, school career, and professional achievements [3]. Psychiatric comorbidities are common and overall quality of life of patients with ADHD is often impaired [4–6]. ADHD also causes significant health costs [7, 8] and represents a major public health problem. Therefore, an effective management of ADHD is needed. In Switzerland, diagnosis and treatment of ADHD are predominantly being provided by adolescent−/child psychiatrists, and pediatricians [9].

In practice, the diagnosis and management of ADHD is complex and involves various difficulties. First, there is no single, specific etiology that is able to explain the emergence of ADHD, and second, no biomarkers exist that can help to clearly establish the diagnosis [10, 11]. Hence, the diagnosis of ADHD is complex and challenging for less experienced physicians. In order to address concerns about the quality of ADHD management and to assist physicians in the diagnosis and treatment process, several guidelines have been developed (e.g. APP American Academy of Pediatrics), CADDRA (Canadian ADHD Resource Alliance), NICE (National Institute for Health and Care Excellence), and the interdisciplinary evidence- and consensus-based (S3) guideline on ADHD in childhood, adolescence and adults [12–15]). However, it is still often criticized that the diagnostic criteria are unclear and the guidelines too vague and not compatible with practice [16–18].

### Diagnostic procedures of ADHD

According to international guidelines, the diagnosis should be based on a comprehensive and structured exploration of the patient and his/her caregivers, including possibly other reference persons (e.g., teachers) in order to assess the frequency and extent of the symptoms and associated impairments in different situations and areas of life as well as comorbid conditions. Questionnaires and psychological tests can assist in establishing a diagnosis in the context of a professional evaluation, which should furthermore be based on a thorough medical history of the child and at least one reference person (e. g., parent, teacher), as well as an assessment of the child including observations and psychological tests [15].

Previous studies examined the diagnostic process of ADHD in children and reported that clinicians perceive the diagnostic assessment of ADHD as challenging [11, 16, 17, 19]. One study, which examines whether child psychologists and psychiatrists in Germany follow the guideline for diagnosing ADHD, has found that therapists did not strictly follow diagnostic manuals, which resulted in an overdiagnosis of ADHD in the presented case vignettes [19]. The authors concluded that overdiagnosis also occurs in clinical practice [19]. Similar, Leitner et al. found that clinicians (i.e., child/adolescent neurologists, − psychiatrists or pediatricians) often adhere to their specific skills when diagnosing ADHD, resulting in differences in the variety and specificity of applied tests and the frequency of performing neurological or physical examinations, which has a significant impact on the perception of the clinical issue [20]. In recent years, concerns about the accuracy and thoroughness of ADHD diagnoses have been raised repeatedly due to the increasing number of children treated with methylphenidate (MPH) [21–23].

Regarding primary care, concerns about the quality of ADHD diagnoses have been raised due to various barriers, such as constraints with diagnosis and treatment, the time-consuming interprofessional work with other specialists, teachers, school social workers and parents, the lack of reimbursement, misconceptions of ADHD among physicians, limited use of diagnostic instruments and consideration of comorbid conditions [16]. However, the high prevalence of ADHD clearly calls for an engagement of primary care providers with regard to diagnosis and treatment of the condition - especially in the light of the limited capacity and the geographically skewed distribution of child/adolescent psychiatrists [14, 24].

### Treatment of ADHD

For the treatment of patients diagnosed with ADHD, the guidelines [15] recommend to first engage in a comprehensive psychoeducation, in which the patient and her/his caregivers are informed about ADHD and different treatment options in order to enable a participative decision about the treatment. In young children and mild forms of ADHD, primary psychosocial interventions are recommended, although dependent on the individual case supplemental pharmacotherapy may be supportive. In moderate to severe forms of ADHD, a multimodal therapy, combining non-pharmacological therapy options (e.g., psychosocial therapy) and pharmacological therapy is indicated. Personal factors and individual predisposition (e.g., level of suffering), the severity of the disorder, environmental factors and any comorbid disorder should be taken into account in the decision whether to delay or start treatment and which therapeutic approach to select [15]*.*

Despite the fact that a multimodal therapy approach for ADHD is considered the “gold standard”, it is unclear to what extent this approach is followed in daily routine. Raising numbers of prescribed pharmacological treatments for ADHD [21–23] have led to concerns that this increase could correspond to a reduced use of other treatments options – particularly since resources for non-pharmacological treatment options are limited in many places. For instance, in Switzerland, in the period 2006 to 2012, the proportion of school aged children (7–15 years) in the canton of Zurich *(overall population 2012: 1.4 million)* who received an MPH treatment within 1 year increased from around 1.5 to 2.4% from 2006 to 2010 [25]. Analyses of health insurance data for the whole of Switzerland (Helsana, Groupe Mutuel, KPT, and Visana) yielded increases of MPH prescriptions of 40% (from 0.61 to 0.85% in children and adolescents [22] from 2005 to 2008, and 42% (from 0.26 to 0.37% in all age groups [21] from 2006 to 2009, respectively. Moreover, the total number of ADHD medications in all age groups prescribed in Switzerland increased by 20.1% between 2017 and 2020 [26]. A study on the prevalence of ADHD across European countries (United Kingdom, France, Germany, Italy, the Netherlands, and Spain) showed that the predominant treatment choice was pharmacotherapy with methylphenidate; about 56% of the studied patients (6–17 years) received MPH, out of which 48.0% were treated with pharmacotherapy only, 34.5% received a combined treatment with pharmacotherapy and behavioral therapy, and 11.6% were treated with behavioral therapy only [27].

Although various studies have described an increase in drug therapies for ADHD compared to other therapeutic measures, reasons for the increase are not entirely clear. Research in social science has indicated that the prevalence of ADHD diagnosis and medical treatment has been influenced by various social factors, such as performance pressure in the school environment [28], gender aspects such as a cultural constructions of masculinity [29–31], or unstable family settings [32]. Even pressure from parents and teachers to prescribe medication has been reported [16]. The question remains whether the guidelines are followed, the diagnosis is carefully clarified, and if multimodal therapy is considered accordingly. This is especially relevant with regard to primary care, which is estimated to account for a substantial proportion of all MPH prescriptions [26].

Building on these recommendations and studies on the practice of ADHD diagnosis and treatment, our study aims to examine Swiss pediatricians’ current practice as well as their perspectives on perceived barriers and facilitators to follow guidelines. Due to the limited number of child and adolescent psychiatrists, pediatricians play a central role in managing ADHD in Switzerland. We therefore aimed at investigating Swiss pediatricians` attitudes and practices regarding ADHD management. The specific study questions were

1. How do pediatricians structure the diagnostic procedure of ADHD (including number of sessions, gathering of information, use of screening instruments)?
2. How do pediatricians approach ADHD treatment and more specifically: do pediatricians apply the recommended multimodal approach?
3. What challenges do pediatricians experience when diagnosing and treating ADHS and what suggestions do they have to improve the management of ADHD?

This first national study of ADHD practices presents comprehensive data regarding the management of children and adolescents with ADHD in pediatric settings in Switzerland.

## Methods

### Data collection

The Swiss Pediatric Society invited all regular and actively practicing members (*n* = 1620, hospital and office-based pediatricians, about 80% of pediatricians of Switzerland) to an online survey (see Table 1) by e-mail using the certified Unipark platform. A subgroup of 65 pediatricians who took part in an advanced multi-day teaching and training courses on ADHD received an additional invitation.

**Table 1:** Questions asked in the survey, arranged according to the three topics

*Diagnostic procedure of ADHD (closed questions)*	
• “For what reasons do the children come to you? Who initiated the assessment?”	
[Responses: never or almost never – rarely – at times – often – always or almost always; cannot say]	
o The child wants it.	
o The parents want it.	
o The school is sending it.	
o The school psychological services sent it.	
o Another person is sending it.	
• “How often do you act as follows if you suspect ADHD during an initial consultation”	
[Responses: 0% - 1-20% - 21-40% - 41-60% - 61-80% - 81-100%; cannot say]	
o I talk with parents about the home, school and wellbeing of the child.	
o I talk to the child about the home, school and his or her well-being.	
o I wait for the explicit wish of the parents before I take action	
o I seek information from additional sources (e.g., school,other environment, ...).	
o I arrange a second appointment, so I can conduct a systematic clarification myself.	
o I refer the patient to a specialized person to carry out a systematic clarification.	
• “How many appointments do you usually schedule for the diagnosis?”	
[Responses: 1, 2, 3, 4, 5, > 5]	
• “How many appointments take place with the child?”	
[Responses: 1, 2, 3, 4, 5, > 5]	
• “Do you use screening questionnaires?” [during the diagnostic process for ADHD] [Responses: Yes, No]	
*Treatment of ADHD (closed questions)*	
• “What information about ADHD do you usually give parents/children when ADHD is diagnosed?”	
[Responses: one check box for each answer, multiple responses possible]	
o I do not provide any further information about the disease.	
o I provide general information about the about the disorder.	
o General brochures on AD(H)S	
o Brochures/information on coaching possibilities, psychological counseling centers, psychotherapy	
o Brochures/information on educational consultations	
o Brochures/information on training possibilities/offers	
o Brochures/information on patient organizations	
o Brochures/information on psychotherapy	
o Brochures/information on drug treatment	
o Brochures/information on the multimodal treatment concept	
o Occupational therapy	
o Family therapy	
o Psychomotor therapy	
o Other information	
• “In case of a diagnosis of ADHD, how often do you inform about the following forms of therapy?”	
[Responses: never – rarely – at times – often – always; not applicable, cannot say]	
o Multimodal treatment	
o Drug treatment	
o Psychotherapy	
o Educational counseling	
o Occupational therapy	
o Physiotherapy	
o Family therapy	
o Psychomotor therapy	
o Others, namely:	
• “How often do you discuss different treatment options with the following people?”	
[Responses: never – rarely – at times – often – always; not applicable, cannot say]	
o Parents	
o Children 4–5 years	
o Children 6–8 years	
o Children 9–11 years	
o Children 12–14 years	
o Teenagers 15–18 years	
o Teacher	
o Other persons, namely:	
• “How does the child’s level of suffering have an impact on your choice of therapy?”	
[Responses: very little –little – moderate – much – very much; not applicable, cannot say]	
• “To what extent do you take the opinions of the following people into account when deciding on treatment?”	
[Responses: very little –little – moderate – much – very much; not applicable, cannot say]	
o Children 4–5 years	
o Children 6–8 years	
o Children 9–11 years	
o Children 12–14 years	
o Teenagers 15–18 years	
o Parents	
o Teacher	
o School psychologist	
o Attending physician (i.e., you)	
o Other persons, namely:	
*Challenges in diagnosis and treatment of ADHD in pediatric practice (open-ended questions)*	
• “Current guidelines provide for multimodal therapy. In your view, what are the three biggest challenges in implementing the multimodal therapy concept?”	
• “In your opinion, what are the three biggest challenges in the diagnosis and treatment of ADHD?”	
• “In which areas would you like more support/further training?”	

In order to achieve coverage throughout Switzerland, parallel language versions of the questionnaire were created in German, French and Italian. The online survey was conducted from January to March 2018 as part of the research project “Supporting children: an interdisciplinary study on the management of ADHD.” that examined how children with attention and concentration disorders can and should be supported [33]. In addition to pediatricians, the project also assessed parents’ and teachers’ perspectives on ADHD using a mixed methods approach [34].

### Participants and sample

Of the 1620 pediatricians who were invited, the starting page of the online questionnaire was visited by 200 participants, out of whom 49 (24.50%) aborted the questionnaire after the first seven questions (16% of the questionnaire) and were excluded from the analyses. Thus, the analysis is based on 151 questionnaires (107 completed, 44 mostly completed), which corresponds to a response rate of 9.3%. Of the 151 questionnaires, 112 participants (74%) completed the German version, 35 participants (23%) the French version and four participants the Italian version (3%). The total of 151 participants consisted of 88 women (58%) and 59 men (39%) as well as four participants with unknown gender (3%). On average, participants had 17 years of work experience (range 4 to 35 years). The majority of pediatricians reported working in private practice (54.1%), followed by collective practice (27%), cantonal hospital (8.2%), university hospital (4.9%), and others (5.8%). Most of the participants were pediatricians (*n* = 122). Other specializations were (child and adolescent) psychiatry and psychotherapy (*n* = 22) and others or unknown specialization (*n* = 7). For the purpose of this study, only the results of the pediatricians are presented in the following. Analyses consist of frequency distributions and Chi-Square tests.

### Statistical analyses

As descriptive analyses, mean and standard values or percentages are presented. As inference statistics, Chi-Square tests were computed to test whether the questions “What information about ADHD do you usually give parents/children when ADHD is diagnosed?” and “In case of a diagnosis of ADHD, how often do you inform about the following forms of therapy?” differ depending on participants’ sex and their years of work experience. To perform the Chi-Square test, years of work experience were aggregated into three equally sized groups (0–17 yrs., 18–23 yrs., and 24–40 yrs.) and the answers to the question how often information is provided about specific forms of therapy was converted to a dichotomous variable (1 = never, rarely) versus (2 = occasionally, often, always).

## Results

### Diagnostic procedure

**Fig. 1:**
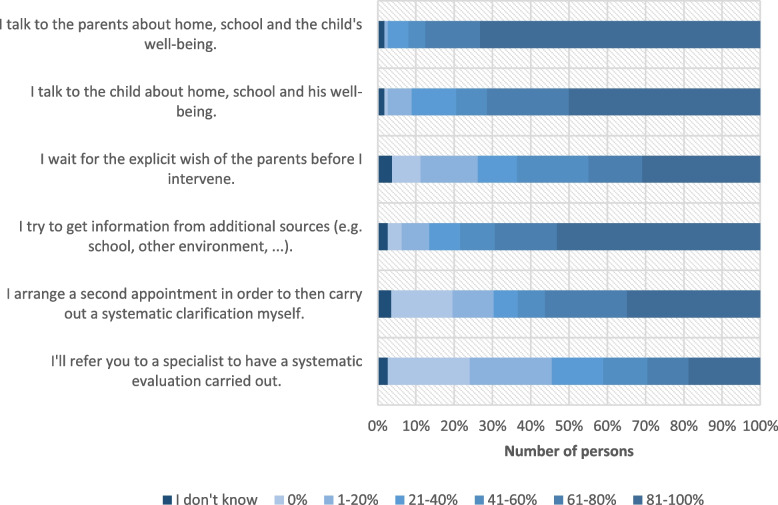
Frequency of actions from pediatricians when ADHD is suspected during an initial interview (*n* = 115), multiple choices allowed

Regarding the question, who initiated the assessment in the first place, the mean ranks indicate that parents (*M* = 1.77, *SD* = 0.87, range = 5–1) and teachers/school (*M* = 1.64, *SD* = 0.72) were rated the most often, while school psychological service (*M* = 2.88, *SD* = 0.78) and the child itself (*M* = 4.41, *SD* = 0.80) were rated less often (see Table 1). When asked, “How often do you act as follows if you suspect ADHD during an initial consultation”, the majority of the participants stated that they almost always (i.e., in 81–100% of all cases; see Table 1) talk to parents about home, school and the well-being of the child (see Fig. 1). Furthermore, doctors mostly or almost always (i.e., in 61–80%, see Table 1) talk to the children themselves and try to obtain information from additional sources (e.g., school), 55% of pediatricians still mostly or almost always arrange for a second appointment to carry out a systematic diagnostic workup themselves and 42% never (i.e., in 0% of cases, see Table 1) or almost never (i.e., in 1–20% of cases, see Table 1) refer to specialized experts for a diagnostic workup.

Pediatricians reported to plan about an average of three (*M* = 3.1, *SD* = 1.06) consultations to establish or rule out the diagnosis with parents and teachers; thereof on average of two (*M* = 2.4, *SD* = 0.97) with the child, when they generally perform the diagnostic workup themselves. The question about the use of ADHD screening instruments for diagnosis (see Table 1) showed that half of the pediatricians use questionnaires for (psychiatric) differential diagnoses, 76% use screening questionnaires (e.g., SDQ - Strengths and Difficulties Questionnaire), and 85% specific ADHD questionnaires (e.g., Conners questionnaire, DISYPS - Diagnostic system for mental disorders according to ICD-10 and DSM-5 for children and adolescents).

### Treatment

**Fig. 2:**
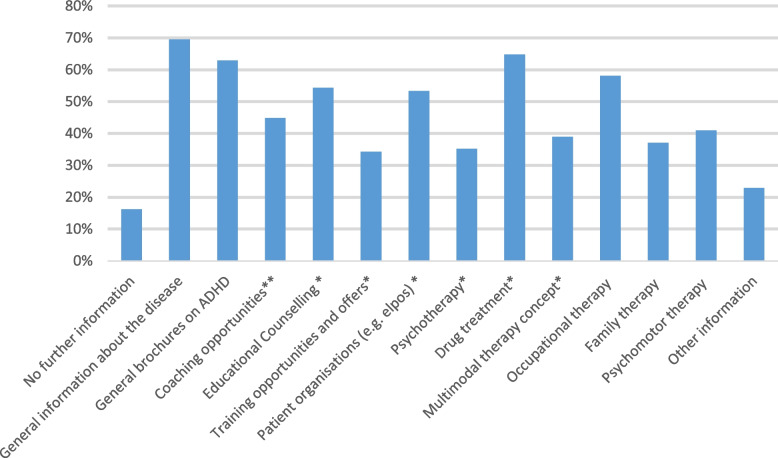
What information do pediatricians give to parents/children when ADHD has been diagnosed? (multiple answers were possible, *n* = 105), multiple choices allowed. Legend. *Information can consist of brochure or other information, **Coaching opportunities include psychological counselling and psychotherapy

With regard to the question, what kind of information pediatricians provide to the families when ADHD has been diagnosed in their child, both general information on ADHD and specific information (e.g., brochures) about coaching possibilities, patient organizations, drug treatment and occupational therapy options were reported to be provided by more than 50% of the pediatricians (see Table 1 and Fig. 2). The option ‘no further information’ was never chosen as the only one, thus, meaning that all pediatricians give at least some kind of information to their patients. All pediatricians who reported to inform about drug treatment also inform about other treatment options. However, frequency distribution of given information did not differ substantially between pediatricians who did or did not inform about drug treatment. Further, what kind of information pediatricians provide to the families did not differ according to years of work experience (*p* = .057), but women tended to give more frequent information on family therapy than men (*p* = .012).

**Fig. 3:**
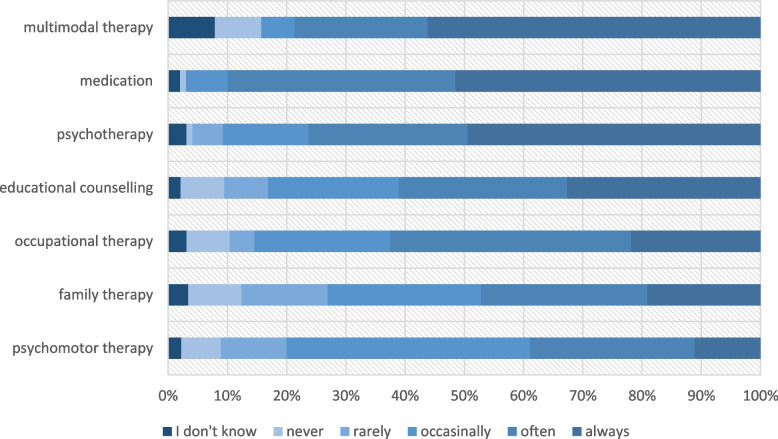
Frequency of information provided by pediatricians about various therapy options when ADHD was diagnosed (*n* = 89–99), multiple choices allowed

When asked how often they inform about different therapy options, the majority (70–90%) reported to always or often inform about pharmacological therapy, psychotherapy, and multimodal therapy (see Table 1 and Fig. 3). Over 50% reported to inform always or often about education counselling and occupational therapy. The frequency of information provided about family therapy and psychomotor therapy varied without a clear trend; 21% of the pediatricians inform never or rarely about family therapy while 42% often or always inform about this treatment option. The frequency with which the therapy options were given did neither differ according to years of work experience (*p* = .177) nor to sex (*p* = .148).

**Fig. 4:**
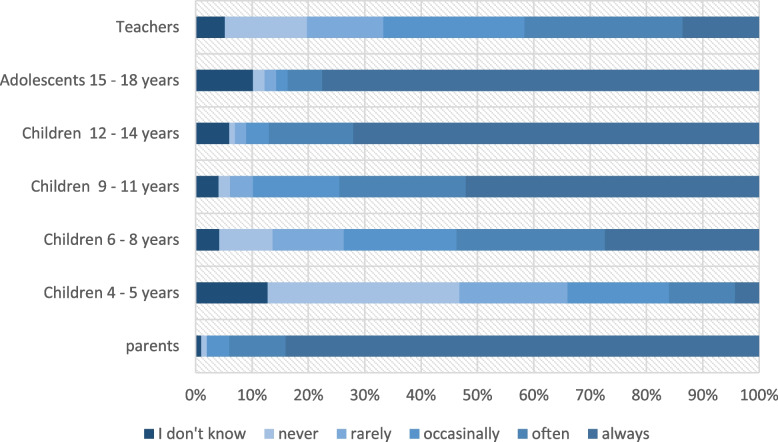
Frequency with which pediatricians discuss treatment options of ADHD with parents, teachers and children in different age groups (*n* = 89), multiple choices allowed

When asked how much the child’s level of suffering plays a role in the choice of therapy, from weak to very strong, 97% of pediatricians consider this to play a strong or very strong role in the choice of therapy. The analysis of the question how often pediatricians discuss treatment options with parents and children of different ages showed that 81% always discuss with parents, and over 90% claimed to discuss them always or often with parents (see Table 1 and Fig. 4). With respect to involving the children in such discussions, nearly half of the pediatricians stated to never or rarely discuss treatment options with young children of four to 5 years of age. From the age of 6 years upwards, however over 50% of the pediatricians often or always involve the child in these discussions. With increasing age of the children, they are more frequently included in the treatment decision. Among adolescents and young adults (15–18 years old), 84% of pediatricians frequently or always discuss treatment options. The frequency with which teachers are included in the discussion varies widely among pediatricians; 28% often include them, while 15% never or always include them. Whereas the opinion of parents is very often (94%) considered in medical decision making, teachers’ opinions are only rarely (42%) taken into account (see Table 1 and Fig. 4).

### Challenges in diagnosis and treatment of ADHD in pediatric practice

In the following, the answers to questions with an open-ended response format are presented categorized into thematic groups. The question “In your opinion, what are the three biggest challenges in the diagnosis and treatment of ADHD?” (see Table 1) was answered by *n* = 81 pediatricians (i.e., filled in at least the first of three empty answer fields), in total, 220 answers were given. Pediatricians most frequently pointed out difficulties in establishing a diagnosis (*n* = 44), including the lack of clarity and completeness of the criteria, variability of symptoms depending on the child, making the right diagnosis, danger of overlooking co-morbidities, assessment of the disposition - environmental influences. Also, very frequent (*n* = 37), the acceptance by the family, involvement of the family, sometimes difficult family constellations or differing opinions in the family regarding treatment were mentioned as challenges. Similarly often were mentioned, the cooperation and involvement of school and teachers (*n* = 17), negative attitudes towards ADHD and explicit negative reactions, for example about the use of ADHD-specific drugs (*n* = 16), do justice to the child (*n* = 15), difficulties in multidisciplinary (e.g., different opinions of professionals and cooperation) (*n* = 12) and good, close supervision during the therapy (*n* = 11), including individual adaptation of the therapy to the child, or finding the right therapy for the individual child and assessment of side effects compared to the benefits.

The question “Current guidelines provide for multimodal therapy. In your view, what are the three biggest challenges in implementing the multimodal therapy concept?” (see Table 1) was answered by *n* = 78 pediatricians (i.e., they filled in at least the first of three empty answer fields), in total, 205 answers were given.

Various reasons were put forward as challenges in the implementation of a multimodal therapy concept: Structural problems were mentioned most frequently (*n* = 57), such as the lack of cooperation and coordination between the professionals involved and the families. Further, the very time-consuming organization, the question of the lead of the organization and the lack of communication between the actors was mentioned. Regarding structural problems, *n* = 9 of the participating pediatricians pointed out the lack of reimbursement options. Frequently, pediatricians pointed out low availability of specialists and therapy places (*n* = 33), particularly the low availability of child and adolescent psychiatrists. In addition to structural/organizational reasons and the lack of professionals, reasons related to the family were often mentioned (*n* = 77). In the context of children and families, their therapeutic adherence and compliance (*n* = 48) was frequently reported as a therapeutic challenge, further the high demand on time and the general lack of capacity on the part of the families (*n* = 16) and lack of motivation (*n* = 5) was mentioned. Some pointed out lack of support and implementation of the concept in the school (*n* = 9) and finally bad or wrong information about the treatment concept, lack of insight and prejudice against medication (*n* = 6) was reported.

The question “In which areas would you like more support/further training?” (see Table 1) was answered by *n* = 68 pediatricians (i.e., filled in the one empty answer field). Pediatricians expressed a need for further training in diagnosing ADHD (*n* = 22) and better support in terms of coordination and cooperation with other specialists (*n* = 7), in particular with teachers and school social workers. More coaching and further training for school professionals was also proposed (*n* = 6), but also the creation of an exchange platform in the sense of a peer consulting (e.g., in groups) (*n* = 3). Finally, stronger efforts were proposed to improve information on ADHD and its treatment in treatment settings (*n* = 5) (e.g., better parent brochures, information material independent of the pharmaceutical industry) as well as in public (e.g., correction of a false, often negative image).

## Discussion

This study provides insights into the pediatric practice and perceived challenges of ADHD management in Switzerland. In accordance with existing guidelines, the majority of participating pediatricians reported to perform a thorough and broad diagnostic procedure with valid diagnostic instruments and involvement of the parents and the child on a regular basis. Pharmacological therapy, psychotherapy, and multimodal therapy were the therapies about which pediatricians informed most often. Reported challenges in the ADHD management were the subjectivity of diagnostic criteria, low availability of psychotherapy options due to a shortage of specialists, and a rather negative attitudes towards ADHD of parents, teachers, and the public. Need for further education for all professionals involved, support for coordination with specialists, and schools and improvement of information on ADHD was expressed. In conclusion, we could not find any evidence for the assumption that participating office-based pediatricians prescribe MPH without embedding pharmacotherapy in a multimodal approach.

### Diagnostic procedures

Acknowledged ADHD guidelines recommend that for the assessment of ADHD more than one visit is scheduled, which is technically difficult within primary care where time is often constrained [13, 18]. On average, pediatricians in this study arrange three meetings with parents for the diagnosis, of which two take place with the child concerned. To our knowledge, there is no published data from other countries for a comparison of how many meetings are in fact held. Parents and schools play the most important part with respect to initiating a diagnostic consultation, while the school psychological service or the children themselves play a less important role. Even though pediatricians report that they assign patients a central role in the diagnostic workup, the child apparently hardly ever initiates the consultation. The inclusion of parents, family members, and teachers in the assessment of ADHD, which has also been found in other studies, is in line with current guidelines [17, 35].

Since the establishment of ADHD guidelines an increased use of screening instruments for the diagnosis has been described in the literature [35]. In line with this, the majority of our participants reported that they use ADHD-specific screening tools. For differential diagnosis, half of our respondents used screening tools.

Pediatricians reported that they perceive the subjectivity of the ADHD diagnosis as challenging, which was also repeatedly reported in other studies [16, 17, 36]. However, only few participants reported to refer children with suspected ADHD to a specialist for a further diagnostic workup. Similar, Gamma et al. observed that pediatricians more often diagnose patients with ADHD themselves compared to general practitioners [37]. In our study, this finding might be accentuated by the fact that pediatricians, who consider themselves competent in this field and see patients with ADHD regularly predominantly participated in the survey. Nevertheless, some participants expressed the need for further training. Another reason for few referrals to specialists could be the shortage of child and adolescent psychiatrists, which was also expressed by our participants and is in line with research from other countries [24, 36, 38].

### Treatment

Once the diagnosis is established, the exchange with parents and children as well as the level of suffering of the child are reported to be central reasons in favoring or deciding against initiating a therapy. Next to drug therapy, pediatricians reported to inform about occupational therapy, educational counselling, and patient organizations most frequently. Most probably, occupational therapy is favored when fine motor impairments are present, which have been described in up to 50% of ADHD patients [39–41].

In our study, involvement of the affected children or adolescents and parents in treatment decisions seems to be standard for many pediatricians and has been reported to be very important for the adherence to therapy [36]. However, pediatricians described the families’ therapeutic adherence and cooperation as a difficulty, they commonly face. This resonates with previous research where despite substantial evidence supporting the efficacy of stimulant medication for children with ADHD, adherence to stimulant treatment was often not optimal [42]. The cause for low adherence was not assessed in the current study. However, proper involvement of the child and family in the therapy process is considered crucial for long-term compliance with therapy [36]. With respect to involvement of the child there seems to be room for improvement. According to the multimodal approach, the majority (80–90%) of pediatricians reported to always or often inform about multimodal therapy, pharmacological therapy and psychotherapy, and the majority (approx. 60%) stated that they always or often inform about education counselling and occupational therapy. This is comparable with the literature, although patients in the study by Venter et al. were more frequently (89%) referred to occupational therapy [37, 39].

Even though pediatricians obviously inform about and apply multiple treatment options, the most frequent therapy still is pharmacological treatment, which corresponds with other findings [37, 39]. Although medication may be prescribed in combination with other therapies, the high use of medication may partly be explained by the reported poor availability of other treatment options such as psychotherapy and occupational therapy. Furthermore, monetary issues for other therapies seem to be a hurdle: for example, occupational therapy was described as problematic with respect to coverage by health insurance companies. An interesting aspect was reported by French et al. which found that physicians felt pressured by parents and teachers to prescribe medication [16].

### Challenges in diagnosis and treatment of ADHD in pediatric practice

Pediatricians reported several challenges in the pediatric ADHD management which mostly correspond with other findings from Switzerland and international studies on ADHD management [16–18, 36, 37]. In a systematic review, French et al. [16] identified four main issues in the management of ADHD in primary care: need for education on ADHD for primary care physicians, misconception and stigma towards ADHD among primary care physicians, constraints with recognition, management, and treatment such as limited time for gathering information from third parties, and issues with the multiprofessional approach, i.e. issues with communication between specialists, schools and parents. In our study, the same issues were found, however, the need for specific education on ADHD and stigmata were only reported by a minority of participants. This might reflect the selection of participants, who predominantly see themselves as competent. Interestingly, while French et al. found stigma and misconceptions among physicians [16], in the current study, participants were referring to stigma in the context of stigmatization of ADHD patients by their environment. However, more education and better information on ADHD for professionals and the public is a finding, which has already been identified in the literature, especially considering the perceived subjectivity of diagnostic criteria and vagueness of guidelines [16, 17]. In our study, pediatricians also suggested further education for school professionals (i.e., teachers and school social workers) and other specialists involved.

Another frequently mentioned issue in our study concerns the multi-professionality of ADHD management, coordination with specialists, schools/teachers and parents, which was described as time-consuming, complicated, and poorly reimbursed. Need for support for interprofessional exchange and case-specific discussions (“patient-centered round tables”) and improvements of reimbursement consequently was frequently stated by our sample and in the literature [16, 17, 35, 39]. Moreover, low availability of psychotherapy places is also a problem that is present not only in Switzerland, but internationally [24, 36, 38].

## Strength and limitations

This first national study on ADHD management presents comprehensive data regarding the management of children and adolescents with ADHD in pediatric outpatient settings in Switzerland. Due to the low response rate, the results should be interpreted with caution, as selection effects may have influenced the results. We most probably reached highly motivated professionals, and pediatricians who treat ADHD in their practice, which already is a selected group. Additionally, all results are based on self-reported data. A non-participation analysis for assessing the reasons of renouncing participation in the survey could not be performed. However, as pediatricians are known to have limited time and resources available, a low response rate was expected.

## Conclusions

Most pediatricians not only invest adequate time in a careful diagnosis of ADHD, but they also follow current diagnostic guidelines by considering a multi-modal approach when treating ADHD and show a high involvement of the family and the child in the choice of therapy options. However, they highlight further potential in the treatment of ADHD by improving the availability of psychotherapy, the cooperation with other specialists, such as teachers and school social workers, and public information on ADHD in Switzerland. Furthermore, better reimbursement of therapies and efforts in the context of interprofessional cooperation with therapists and schools were proposed. Various efforts directed at physicians involved in the care for ADHD patients, therapists, politicians, schools, and families emerged to be relevant 1.) to inseminate sufficient know-how about diagnostics and treatment to primary care providers, 2.) to install adequate reimbursement tools for covering interprofessional work for patients with ADHD, 3.) to improve the availability of non-pharmaceutical therapies and support for ADHD in order to support a sustainable adherence with the recommended guidelines.

## Data Availability

The data presented in this study are available on request from the corresponding author. The data are not yet publicly available due to legal and privacy issues.

## Abbreviations

ADHD: Attention deficit/hyperactivity disorder
APP: American Academy of Pediatrics
CADDRA: Canadian ADHD Resource Alliance
NICE: National Institute for Health and Care Excellence
MPH: Methylphenidate
